# Design and preliminary validation of a high-fidelity vascular simulator for robot-assisted manipulation

**DOI:** 10.1038/s41598-024-55351-8

**Published:** 2024-02-27

**Authors:** Giulia Gamberini, Sabina Maglio, Andrea Mariani, Alessandro Dario Mazzotta, Antonello Forgione, Jacques Marescaux, Franca Melfi, Selene Tognarelli, Arianna Menciassi

**Affiliations:** 1 Health Science Interdisciplinary Center, Scuola Superiore Sant’Anna, Pisa, Italy; 2 The BioRobotics Institute, Scuola Superiore Sant’Anna, Pontedera (Pisa), Italy; 3 The Department of Excellence in Robotics & AI, Scuola Superiore Sant’Anna, Pisa, Italy; 4 Department of Surgery, Madre Giuseppina Vannini Hospital, Istituto Figlie Di San Camillo, Rome, Italy; 5 IRCAD France, Institut de recherche contre les cancers de l’appareil digestif, Strabourg Cedex, France; 6 University of Pisa, Pisa, Italy

**Keywords:** Biomedical engineering, Gels and hydrogels

## Abstract

The number of robot-assisted minimally invasive surgeries is increasing annually, together with the need for dedicated and effective training. Surgeons need to learn how to address the novel control modalities of surgical instruments and the loss of haptic feedback, which is a common feature of most surgical robots. High-fidelity physical simulation has proved to be a valid training tool, and it might help in fulfilling these learning needs. In this regard, a high-fidelity sensorized simulator of vascular structures was designed, fabricated and preliminarily validated. The main objective of the simulator is to train novices in robotic surgery to correctly perform vascular resection procedures without applying excessive strain to tissues. The vessel simulator was integrated with soft strain sensors to quantify and objectively assess manipulation skills and to provide real-time feedback to the trainee during a training session. Additionally, a portable and user-friendly training task board was produced to replicate anatomical constraints. The simulator was characterized in terms of its mechanical properties, demonstrating its realism with respect to human tissues. Its face, content and construct validity, together with its usability, were assessed by implementing a training scenario with 13 clinicians, and the results were generally positive.

## Introduction

The number of robot-assisted minimally invasive surgical procedures is increasing annually^1^, as is the demand for specialized and effective training platforms. Currently, the most successful example of a surgical robotic system is the *da Vinci surgical system* (Intuitive Surgical, Inc., CA, USA)^2^. Despite the well-recognized advantages of surgical robotics, the main drawback of the da Vinci surgical system (and, in general, of the vast majority of robotic surgical devices) is the complete lack of haptic feedback^3^. In surgery, haptic or force feedback refers to the sense of touch the surgeon experiences while performing a procedure^4^. A lack of force feedback may prolong learning curves and operative times, increasing the risk of surgical errors^4^. Consequently, extensive training is needed to avoid the risk of improper use of surgical robots. In several surgical procedures, such as lung lobectomy^5,6^, liver resection^7,8^, or more generalized tumor resection^9^, the application of a mechanical stapler to major blood vessels is required to stop the vascularization of the target tissue to be removed. During these tasks, the most common error is excessive tension^10^, which can lead to rupture of the blood vessel and severe bleeding and is the major cause of conversion from minimally invasive to open surgery^11^. In addition, interventional bleeding is associated with a longer hospital stay and a higher incidence of other postoperative complications^11^. In robotic surgical procedures, surgeons try to compensate for the lack of haptic feedback by using 3D vision to determine the amount of tension and traction applied to tissues in the different phases of the procedure. On the other hand, this compensation ability largely depends on surgeon experience, and training has been demonstrated to be crucial for gaining experience in a safe way^5,11,12^. Training allows surgical conditions to be replicated, providing technical and nontechnical capabilities to the surgeon, with the final aim of reducing errors and intraoperative adverse events, thus increasing patient safety. Surgical simulations specifically aid in the development of critical psychomotor, technical and judgment skills; reduce operative times; lower the risk of complications; and improve patient outcomes^13^.

During the last twenty years, the high-fidelity organ simulation field has received increasing interest from the scientific and clinical communities^14^. A high-fidelity physical simulator should reproduce the correct anatomy, employ materials that replicate the biomechanical properties of human tissues and simulate the behavior of real organs during surgery^14^. Thus, the ideal simulator for robotic surgical training should be able to reproduce, with high fidelity, the target anatomy, the mechanical properties of the tissues it is intended to replicate and the robot-tissue interactions. Moreover, the simulator should provide an objective assessment of users’ skills to allow trainees to reach their maximum of their learning curves^15^.

Our clinical partners expressed the need for training novices in tasks in which vascular structures need to be addressed, as this occurs during lung lobectomy because of the delicate nature of the involved tissues and the high rate of conversion to open surgery due to intraoperative bleeding^16^. For these reasons, lung lobectomy was chosen as the target procedure, and the pulmonary vein was selected as the target anatomy. According to the literature, few sensorized *low-fidelity* physical simulators of vascular structures are available. These devices simulate different surgical tasks, such as suturing, vessel ligation or endovascular navigation^17–19^. The evaluation methods used to assess these kinds of sensorized simulators (i.e., those equipped with force sensors, optical sensors, etc.) are based on the interaction between the surgeon’s tools and the simulated patient’s tissue^20^. However, physical *high-fidelity* vessel simulators are not as common in the literature, and very few examples of such simulators exist^21–23^. In their work, Morikawa et al.^21^ presented a real-size chest model equipped with soft structures that replicates two vessels: the trachea, bronchi, and lung parenchyma. The pulmonary vessels were casted using soft rubber. Neto et al.^22^ constructed a chest simulator with 3D-printed lungs for use as a pulmonary suturing model for training surgeons who perform video-assisted thoracoscopic surgery (VATS). The vascular structures of this model were manufactured with a thermoplastic elastomer. Štupnik et al.^23^ presented a simulator for training minor and major resections via VATS. The pulmonary vessels were made of polyvinyl chloride.

The main drawback of the sensorized simulators presented above^17–19^ (i.e., low-fidelity simulators) is the lack of realism in terms of their mechanical properties and anatomical structures. Regarding physical high-fidelity simulators^21–23^, their main drawback is the lack of sensorization, which prevents these systems from being used to objectively evaluate trainee performance and, in addition, to provide feedback to users. Indeed, feedback has been extensively demonstrated to be a fundamental component of learning optimization^24^.

To overcome the drawbacks of state-of-the-art simulators, we present the optimization and validation of a prototype^25^ that matches the specifications of an ideal simulator. Although generally well received, the preliminary prototype was considered by clinicians to be inadequate for a fully realistic simulation. Rather than a vessel lying on a plastic board, the optimized prototype consists of a vessel simulator housed in its soft surrounding tissue with the aim of resembling the adipose tissue vessels are usually covered with. It should be noted that proper artificial tissue that allows electrocauterization is needed to simulate correct vessel isolation. The mechanical properties of the vessel and the surrounding tissue were tuned to replicate the same structures and ex vivo porcine vein described in the literature. Due to the unavailability of the commercial material originally used, extensive effort has been invested in developing a new and customized sensor solution along with a new sensor integration technique to enhance the reliability of the sensor’s signal readings. Improved portability of the platform and quick and intuitive replacement of the simulator components were successfully achieved. Furthermore, more attention has been given to ensuring stable fixation of the task board to a standard surgical table to improve the overall reliability and reproducibility of the testing sessions. The final prototype is then validated in terms of its face, content and construct validity by involving a pool of surgeons with heterogeneous levels of experience in robot-assisted surgery (RAS).

## Methods

In this section, the methodologies used to design, fabricate and sensorize the proposed simulator are presented. In addition, the mechanical characterization of its components (i.e., vessel and soft surrounding tissue) is described. The electronics and the software implemented to obtain a graphical user interface (GUI) to interact with the simulator are detailed. In conclusion, the validation process (performed with clinicians) is discussed.

This study was approved by the IRCAD INTERNAL COMITEE ETHIQUE, IRCAD-ICOMETH, (protocol: “IRCAD—New Devices 2022–2025”) that has the allowance of approving studies involving human participants. The current study was carried out at the IRCAD (https://www.ircad.fr/) facilities (Strasbourg, France). All the experiments were performed in accordance with the General Data Protection Regulation (GDPR). All the recruited surgeons signed an informed consent form before starting the experiments.

### Target task to simulate

**Figure 1:**
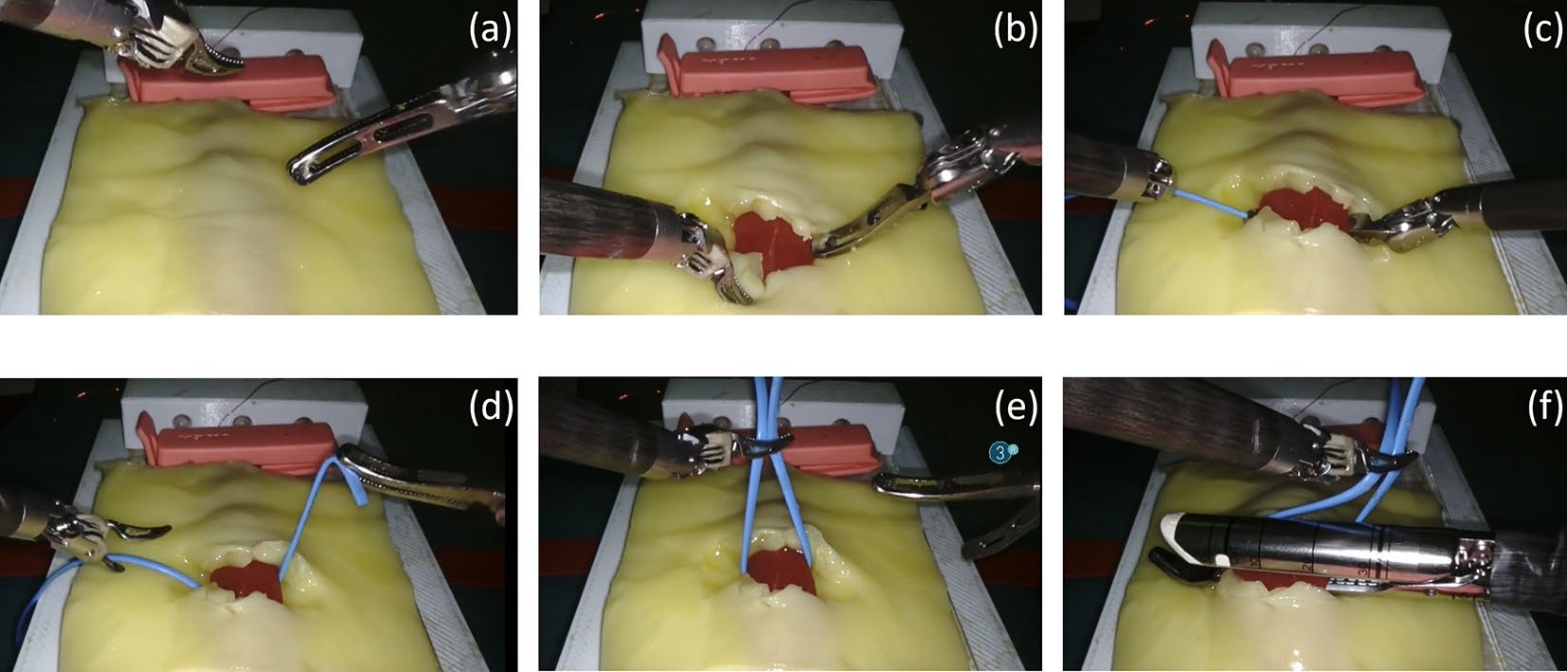
Outline of the target surgical procedure. At the beginning, the tool on the left was Maryland bipolar forceps, and the tool on the right was a tip-up fenestrated grasper. (**a**) Vessel isolation: removal of the adipose tissue surrounding the vessel. (**b**) Exposure of the vascular structure: a window in the adipose tissue is made. (**c**) Insertion of the tip-up fenestrated grasper under the vessel. (**d**) Passage of the vessel loop below the vessel. (**e**) Lift of the vein using the vessel loop. (**f**) Insertion and closure of the stapler (right instrument). The fenestrated grasper substitution with the stapler is not represented because it is performed by an external surgical technician between steps (**e,f**).

The proposed simulator was designed to reproduce the isolation and resection of the pulmonary vein during lung lobectomy. The main steps of the target procedure, as shown in Fig. 1, are as follows: vessel isolation, that is, removal of the adipose tissue surrounding the vessel (Fig. 1a); vessel exposure through the realization of a window into the adipose tissue (Fig. 1b); insertion of the tip-up fenestrated grasper (instrument shown on the right) (Fig. 1c); vessel loop passage (blue strap) below the vessel (Fig. 1d); lift of the vein by using the vessel loop (Fig. 1e); and insertion and closure of the stapler (Fig. 1f)^26^. Between the last two steps, the right instrument is changed by a surgical technician. The tool on the left (i.e., Maryland bipolar) is the same for all steps of the task. A video of the procedure performed by an expert surgeon is provided in the Supplementary Information.

### Design and fabrication of the physical simulator

**Figure 2:**
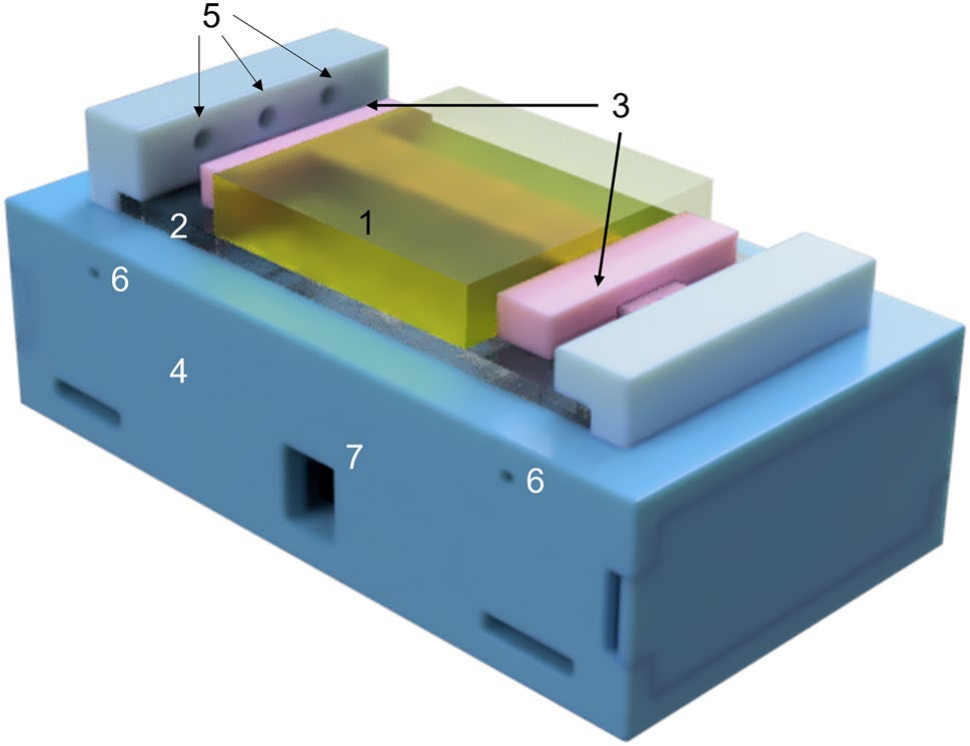
Final design of the simulator realized using Fusion 360 software. Main components: 1. Surrounding tissue housing the vessel; 2. Acrylic sheet; 3. Anchoring systems; 4. Support structure of the simulator with connectors; 5. Three LEDs housing; 6. Hole for cable routing; 7. Hole for the power supply.

The main anatomical components of the simulator are the vessel and the surrounding tissue where it is embedded (Fig. 2(1)). The vascular structure was simulated as a soft cylindrical tube whose dimensions were tuned with respect to the anatomical one^27^, while the artificial soft tissue was designed to completely cover the vessel. The pulmonary vein was chosen as the anatomical target to be replicated. Thus, the dimensions reported in the literature^27–29^ and replicated in the design of the vascular structure are as follows: length of $120\text{ mm}$, diameter of $13\text{ mm}$ and wall thickness of $1.50\text{ mm}$. The surrounding tissue was designed as a parallelepiped ($80\text{ mm}\times 80\text{ mm} \times 16\text{ mm}$) with an elliptic central hole for vessel housing, allowing us to replicate the anatomy. The elliptical shape was chosen because the vein takes that shape when locked in the anchoring systems. A support structure was also designed and constructed to improve the usability and portability of the simulator (Fig. 2(4)).

To realize the anatomical components of the simulator, molds were designed with Fusion 360 software (Autodesk, CA, USA) and 3D printed with an Original Prusa i3 MK3s + 3D printer (Prusa Research, Czech Republic). The mold of the vein was realized in polylactic acid (PLA), while for the mold of the surrounding tissue and for the support structure, polyethylene terephthalate modified with glyco (PETG) was employed.

Silicone Ecoflex 00-30 (Smooth-On, USA) was used for vein simulator casting; part A and part B of the silicone were mixed 1A:1B by weight. Ecoflex 00-30 provides good compromise in terms of its flexibility, softness and structural integrity; moreover, it is recognized in the literature to have mechanical properties similar to those of human soft tissues and muscles^30^. Polyvinyl alcohol (PVA) was used to create soft surrounding tissue because it resembles the mechanical properties of the adipose tissue that physiologically covers the vein. PVA is a water-based cross-linked polymer whose mechanical properties can be tuned by using a freeze‒thaw process to closely match those of human soft tissues^31^. In addition, PVA is electroconductive, thus enabling the use of electrocautery or other energy device^32^ for vessel isolation tasks, as is commonly done during surgery. For the soft surrounding tissue, we chose $4\mathrm{\% }w/v$ (4 g of PVA in 100 ml of distilled water) PVA because it better replicates the mechanical properties of human tissue (according to indications noted in the literature)^31^. To define the optimal number of freeze‒thaw cycles that would result in synthetic adipose tissue with mechanical properties close to those of real adipose tissue, three samples were collected. The first sample was subjected to one freeze‒thaw cycle, the second to two freeze‒thaw cycles and the third to three freeze‒thaw cycles. The duration of each phase of the freeze‒thaw cycle was 8 h. Consequently, the samples were initially placed in the freezer for 8 h, followed by 8 h at room temperature. The three samples were tested by twelve surgeons, who assessed their similarity to real adipose tissue via tactile handling. The sample that had undergone three freeze‒thaw cycles was deemed to be the most similar to the adipose tissue that surgeons usually face in clinical practice. To respond to general sustainability needs, the vein has been designed to be reusable because it is not necessary to activate the stapler at the end of the procedure, while the surrounding tissue has been designed to be disposable because it has to be physically damaged during training. The task was considered completed when the surgeon, after the stapler insertion, closed it—see Fig. 1f for reference.

**Figure 3:**
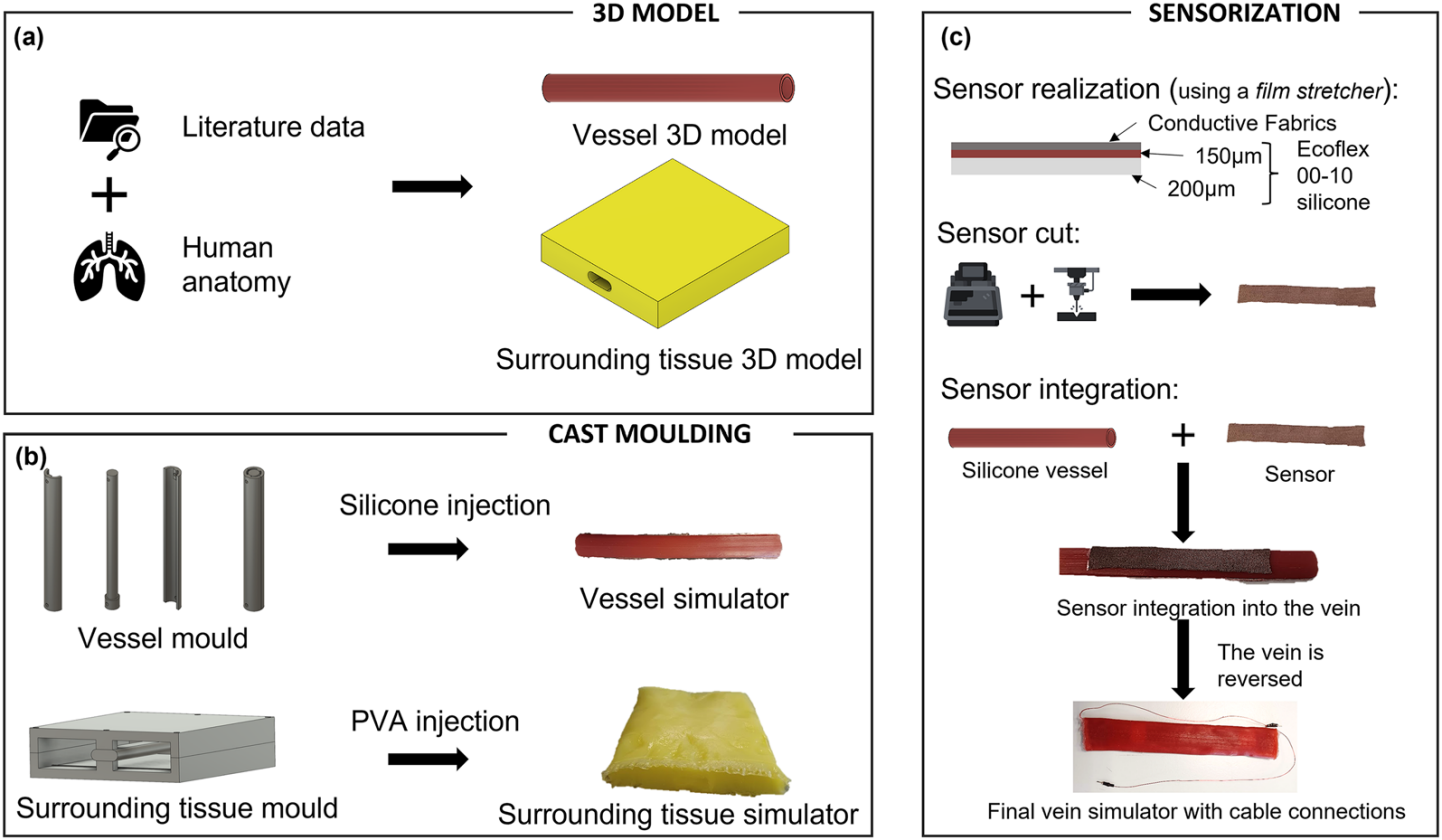
Simulator manufacturing process. (**a**) CAD model of the anatomical structure components of the simulator. (**b**) Cast molding of the different components. (**c**) The sensorization process: sensor realization, sensor cutting and sensor integration.

The CAD models of the simulator components and the main fabrication steps are shown in Fig. 3a,b, respectively.

The support structure was designed using Fusion 360 software as a box ($198\text{ mm} \times 106\text{ mm} \times 54\text{ mm}$) equipped with dedicated anchoring mechanisms (Fig. 2(3)) used to easily fix the vein embedded in the surrounding tissues and three LED housings for visual user feedback during the training session. The electronic components are placed in the internal portion of the box. On the bottom part of the box, four rectangular holes (two for each side) are designed to let two luggage straps with bushings be passed to firmly attach the simulator to the surgical table. Holes for cable routing and power supply are also included (Fig. 2(6),(7), respectively). The final design of the whole structure, simulator components and support box are presented in Fig. 2.

It is worth mentioning that the two anchoring systems are used to easily lock the vessel at the support structure and make the simulator “plug and play”. Moreover, the anchoring systems are glued to an acrylic sheet for easy maintenance (Fig. 2(2)); in the case of anchoring system breakage, replacing the acrylic sheet is sufficient for restoring the simulator functionalities.

In this way, the vascular structure can be changed very easily without the need for any technician, simplifying its future translation in the clinical environment.

### Sensorization

Since the most common error during vessel manipulation during RAS is the exertion of excessive tension^10^, deformation of the vascular structure is chosen as an evaluation metric in a training program. To measure vessel elongation, a stretching sensor is the elective solution for the sensorization of the vein. As we want the sensor to be integrated into the vessel simulator without causing a loss of realism, it must be soft, integrable in silicone and able to follow the deformation of the vascular structure without altering the mechanical properties of the structure itself. Flexible commercial sensors (i.e., ElastiSense) were not selected because they require stretching forces (120 N) higher than those involved in our application. Moreover, the integration requirements are not met, and the measurement area cannot be finely tuned. Based on the above considerations, a resistive customized stretching sensor was chosen. The sensor was realized starting from Stretch Conductive Fabric tissue (Less EMF, NY, USA), a conductive elastic fabric made of $76\%$ nylon and $24\%$ elastic fibers that can be easily shaped and integrated into a soft structure. Moreover, conductive fabrics have been demonstrated to represent a valid technological solution in many applications, such as wearable technologies^33^, monitoring biomedical parameters^34^, soft robotics and human–robot interface design^35^.

Resistive stretching sensors are characterized by a change in electrical resistance when the sensors, and consequently the structures in which the sensor is integrated, undergo a deformation process. Considering the specific application purposes of this study, the sensor was designed and fabricated to fit the entire region of interest used by the surgeon during the procedure. However, to correctly integrate the sensor into the vascular silicone structure and guarantee robustness and precision during the simulation, the layer of silicone must be equally distributed on the whole surface of the sensor to avoid any changes in the sensing properties. To achieve this, a film stretcher (TCQ Sheen B. V., Netherlands) was used. A thin layer (200 μm) of silicone Ecoflex 00-10 (Smooth-On, USA) was cast onto a PETG sheet and cured in an oven at 50 °C for 10 min; then, another layer, 100 μm in height, was added on top of the previous layer, and the fabric was then positioned on top. Oven curation was performed at 50 °C for 15 min. The final fabric-silicone sensor was shaped using a laser cut machine (Versalaser, Universal Laser Systems, Inc., Arizona, USA) by using $13\%$ power, $15\%$ speed and 3 mm height as working parameters. The cut sensor was then integrated into the vein by using the film stretcher again. A thin layer, 150 μm in height, of Ecoflex 00-10 silicone was added to the top surface of the silicone vessel, and the cut sensor was carefully positioned on top. The silicone was left to cure at room temperature for 4 h.

Signal acquisition from the sensorized vessel was performed by means of electrical connections with conductive wires attached to the sensor surface. The conductive wires and the pins were welded together, and insulation was obtained using heat shrink. A schematic view of the vessel sensorization process is shown in Fig. 3c.

### Electronics and software

**Figure 4:**
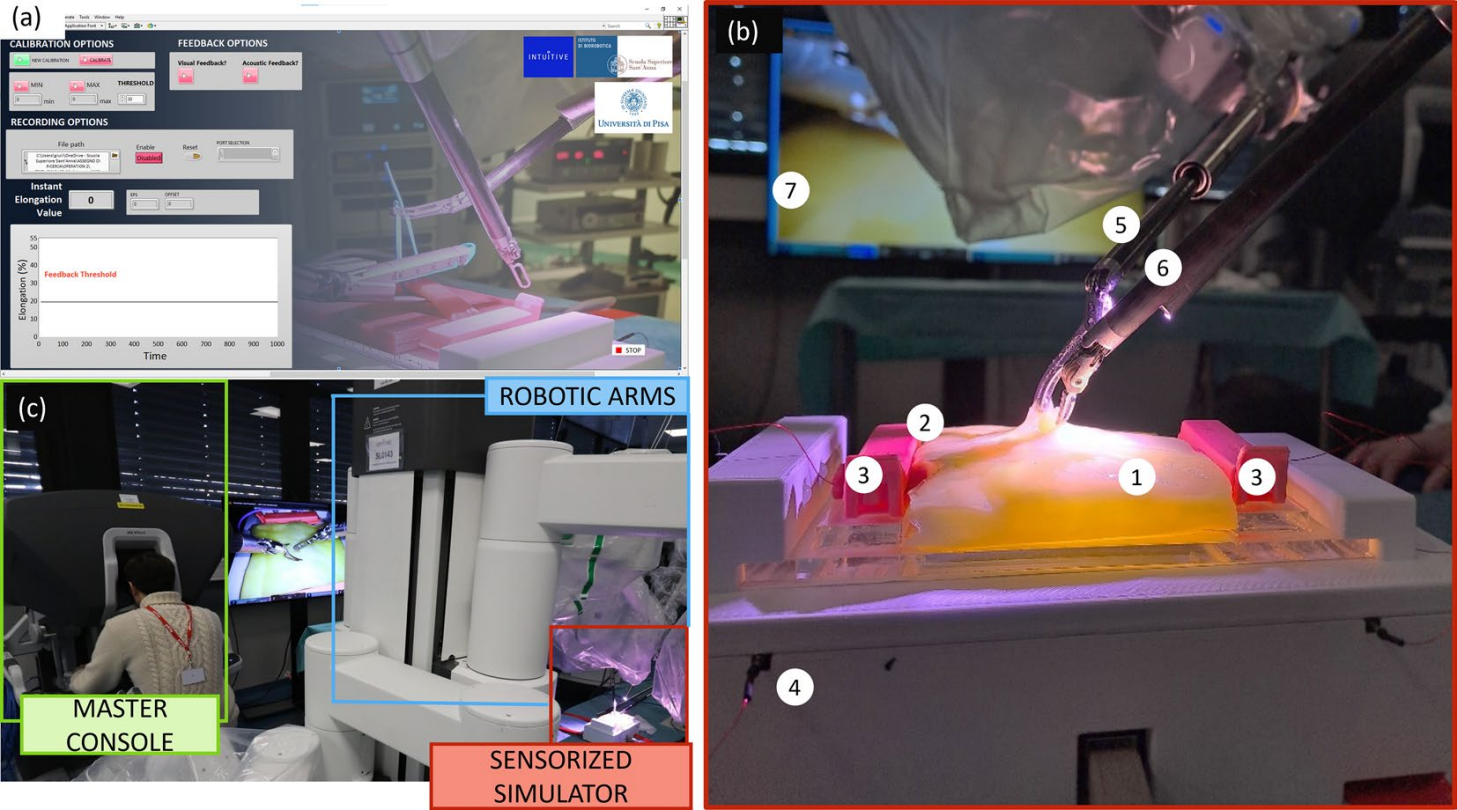
Graphical user interface, final assembly of the simulator under the da Vinci X and experimental setup during the tests performed at the IRCAD Centre (Strasbourg, France). (**a**) The graphical user interface used during the tests. (**b**) A zoom-in image of the simulator. In particular: 1. PVA-based surrounding tissue that resembles human adipose tissue, 2. part of the vein simulator housed in the adipose tissue, 3. the two anchoring systems, 4. the support box, 5. the tip-up fenestrated grasper, 6. Maryland bipolar and 7. monitor showing the images taken from the endoscopic camera. (**c**) The experimental setup of the whole test with the surgeon at the master console of the da Vinci robot and the simulator on the surgical table.

Ad hoc electronic components for signal conditioning and acquisition were integrated into the training board, and a GUI was designed and implemented to control and manage the simulator during training. The electronics were designed using Fritzing software (Interaction Design Lab della Fachhochschule Potsdam, Germany), with the final aim of obtaining reliable sensor data and providing real-time feedback to the users. An Arduino Uno control board (Interaction Design Institute Ivrea, Italy) and a Wheatstone bridge were used to measure the electrical resistance of the stretching textile sensor. A buzzer and three LEDs were also added to the electronic circuitry to provide real-time feedback to the users during training. When the value read by the sensor overcomes a predefined threshold (e.g., defined by the proctor or based on data from experts), acoustic and visual feedback can be activated (individually or both). To control the electronics embedded in the simulator and to design the GUI (Fig. 4a), LabVIEW software (National Instrument, USA) was used. The data were acquired at a sampling frequency of 35 Hz.

### Mechanical characterization

The mechanical properties of the sensorized vessel simulator were evaluated through cyclic monoaxial tensile tests performed with an Instron machine (Instron, USA). From the tensile tests, both the force‒displacement and sensor-value-displacement curves were acquired. Three cyclic monoaxial tensile tests with 3 up-and-down cycles, from 0 to 40% of elongation at a rate of 200 mm/min, were performed. The rate was arbitrarily chosen not having clinical and literature references to use. The decision to perform the tests from 0 to 40% of vessel elongation derives from the vein rupture data available in the literature, as reported by Cabrera^36^: the percentage of deformation at which vein breaks occur is 30%. To compare the mechanical properties with those of real anatomical structures, tensile tests were performed on fresh porcine pulmonary veins and arteries obtained from a slaughterhouse, and the results were also compared with published data from a porcine artery^37^.

For the mechanical characterization of the PVA-based surrounding tissue, we performed compression tests. These tests were performed to compare the Young’s modulus of the human adipose organ-surrounding tissue and the PVA-based artificial tissue. Five up-and-down cycles, controlled by a displacement percentage ranging from 0 to 90% at a rate of 200 mm/min, were performed with an Instron machine.

### Validity and usability assessment

The validation of the simulator was carried out by considering both data collected from dedicated surveys and the data obtained from the integrated sensor. The assessment of the simulator was divided into two phases: face and content validity and usability, as assessed by means of surgeons’ surveys; construct validity was investigated considering the data obtained from the sensor output during tests performed by clinicians.

*Face validity* is defined as the extent of a simulator’s realism and appropriateness when compared to the target task. Face validity addresses the question of how realistic a simulator is^38^. *Content validity* is defined as the extent to which a simulator’s content is representative of the knowledge or skills that must be learned in the real world^38^. *Construct validity* determines whether the simulator can differentiate between heterogeneous levels of expertise. Construct validity is usually used to evaluate the testing instrument quality or ability it was designed to measure^39^. The *usability* of the simulator refers to its user friendliness, simple setup and ergonomics^40^.

#### Experimental setup

The experiments were carried out at the IRCAD training center (Strasbourg, France) using the da Vinci Surgical System X (Intuitive Surg. Inc., CA, USA). A tip-up fenestrated grasper was mounted on the right arm of the robot, and Maryland bipolar forceps were mounted on the left arm. During the simulation, after vessel loop passage, the tip-up fenestrated grasper was changed (by a bedside assistant) with a stapler 30 to guarantee the best working conditions from the beginning to the end of the simulation procedure.

#### Acquisition protocol

The simulator, in all its components, was presented to 13 surgeons. Before starting the experiments, the validation protocol was presented to the end users through a slide presentation. An analysis of functioning of the active simulator and a brief description of the main features of the graphical user interface were carried out. A video of the entire surgical task performed by an expert surgeon was used as a reference for testing. The surgeons were asked to perform two repetitions of a simple retraction task without the PVA-based tissue to familiarize themselves with the simulator setup. After familiarization, they were asked to perform two entire repetitions of the complete vessel isolation task. The surgeons were asked not to staple the vessel to avoid wasting materials. No feedback (neither visual nor acoustic) was provided to the user during the task. The sensor data were recorded to evaluate the construct validity of the simulator. Before the beginning of each session, users were asked to sign an informed consent form.

#### Evaluation

At the end of the test, surgeons were asked to complete a survey that included questions concerning both face and content validity, as well as system usability (the proposed questionnaire is available in the Supplementary Information). The users answered by using the Likert scale as a reference; thus, they answered on a scale ranging from 1 and 5, where 1 means strongly disagree and 5 means strongly agree. The questions about face validity were related to (i) the overall global impression of the system, (ii) the anatomical structures and their visual realism and (iii) the mechanical and haptic response by handling the anatomical structures with their hands and during a tissue-instrument interaction. For content validity, surgeons were asked to evaluate the usefulness of the system in teaching vascular structure isolation and minimization of the applied forces. To evaluate construct validity, the recorded data were analyzed to compare the performance with respect to the surgeons’ experience level. The group of surgeons employed for the analysis of construct validity was divided into experts and nonexperts. Surgeons with more than 3 years of experience in RAS as the first operator were considered experts. The expert group consisted of two professors with more than 20 years of experience in laparoscopic surgery and 10 to 20 years of expertise in RAS. The group also included three expert surgeons with more than 5 years of work in laparoscopy and between 3 and 20 years as the first operator in RAS. Conversely, the nonexpert group included two specialists with 5 to 10 years of experience in laparoscopic surgery but lacked proficiency as a first operators in RAS and three residents in their first to fourth years of specialization, with no prior experience with RAS. The metric used to analyze the data is the maximum percentage of deformation applied to the vessel during task execution. This metric was chosen because one high peak of strain should be considered dangerous. In the real surgical scenario, the decrease in the maximum strain applied to vessel structures may prevent the onset of intraoperative events caused by major bleeding. A statistical analysis was performed to assess any significant difference between expert and nonexpert users. Due to the sample size, a nonparametric statistical significance test, i.e., the Mann‒Whitney U test, was used to evaluate significant differences in median performance. A statistically significant effect was defined as a p value < 0.05. The statistical analysis was performed in MATLAB using the command *ranksum()*. The usability of the simulator was analyzed according to the System Usability Scale (SUS), a standard tool used to quickly test the ease of use of a device created by John Brooke^40^. All the surveys were administered anonymously, and no personal or sensitive information were asked.

## Results

### Assembly of the simulator

The final prototype of the smart vessel simulator is presented in Fig. 4b. The simulator is composed of two main anatomical structures, i.e., the vessel (in red) and its surrounding tissue (in yellow). These anatomical structures are placed on the simulator base and fixed by means of two anchoring systems. The electronic components are placed inside the support structure to obtain a portable and easy-to-use training system.

### Mechanical characterization

**Figure 5:**
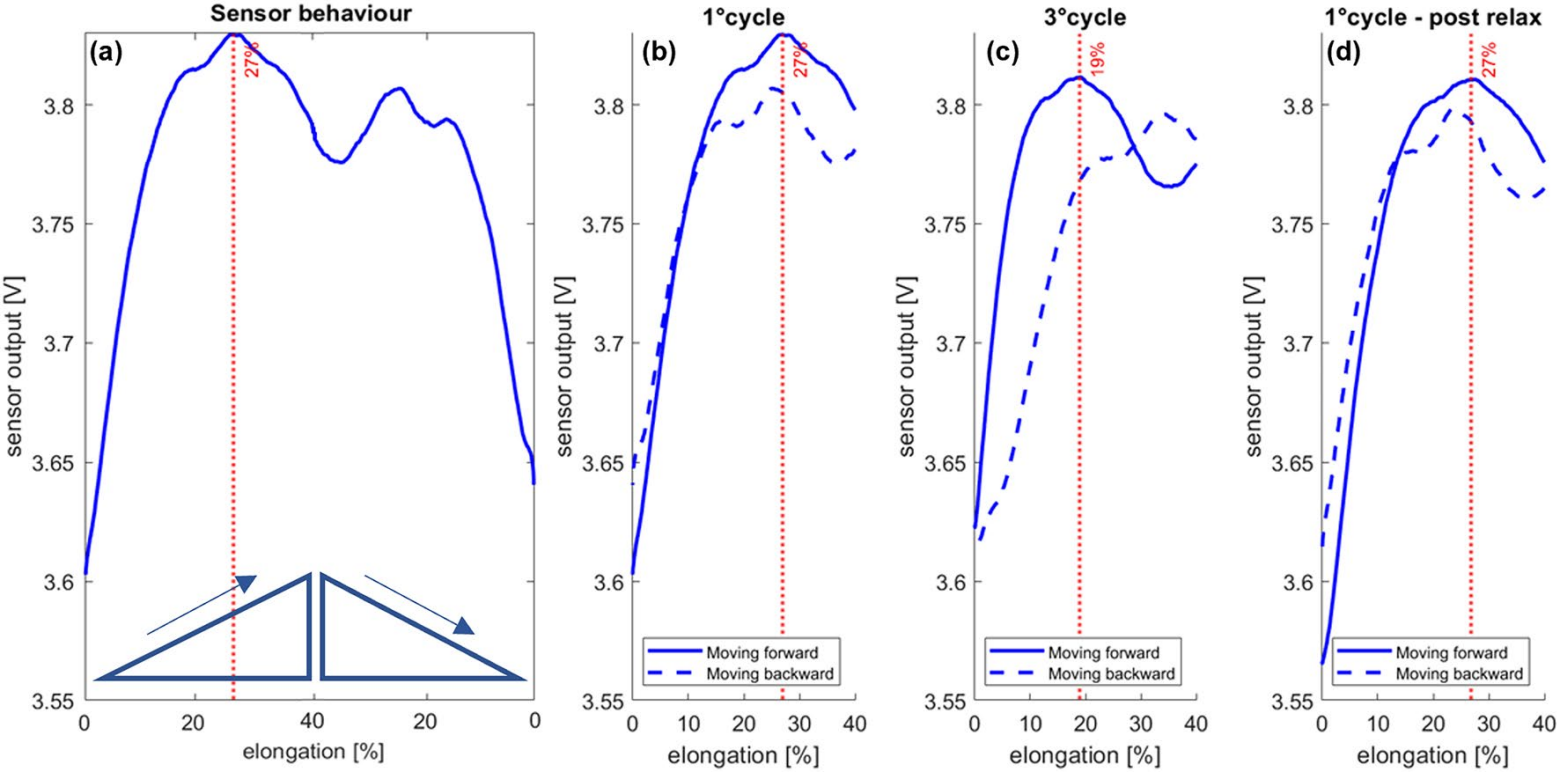
(**a**) Sensorized vein sensor output during the cyclic mechanical characterization tests. The elongation was between 0 and 40%. The sensor exhibited a typical “m shape” behavior. The red dotted line, corresponding to 27% deformation, highlights the peak value of the sensor output. (**b**) Sensor output-elongation curve during the first cycle of the tensile test. (**c**) Sensor output-elongation curve during the third cycle of the tensile test. (**d**) Sensor output-elongation curve during the first cycle of the tensile test after a relaxation period of 3 min.

Regarding the *vessel mechanical characterization*, the sensor output behavior during the cyclic tensile test is shown in Fig. 5a. The sensor shows an “m shape” behavior in the investigated range with a change in the curve slope at approximately 27% of elongation. This result is satisfactory for our application considering that a real vein could break beyond 30% of stretching^36^. As shown in Fig. 5b, the sensor responds to stretching with hysteresis in the first cycle and an additional relaxation phenomenon in the third cycle of testing (Fig. 5c). After 3 min of rest, the sensor returns to its initial state (Fig. 5d), showing the peak of its curve at the same percentage of elongation as in the first cycle. This behavior is typical of viscoelastic materials, and being the conductive fabrics made of 24% of elastomers, the obtained results are in line with the literature^41^. In the framework of the training course, the relaxation phenomenon should not be considered an issue. In fact, as the PVA tissue is disposable, it must be changed between one repetition of the task and the following. Thus, there is always three minutes of time for the vein to restore to the normal behavior.

**Figure 6:**
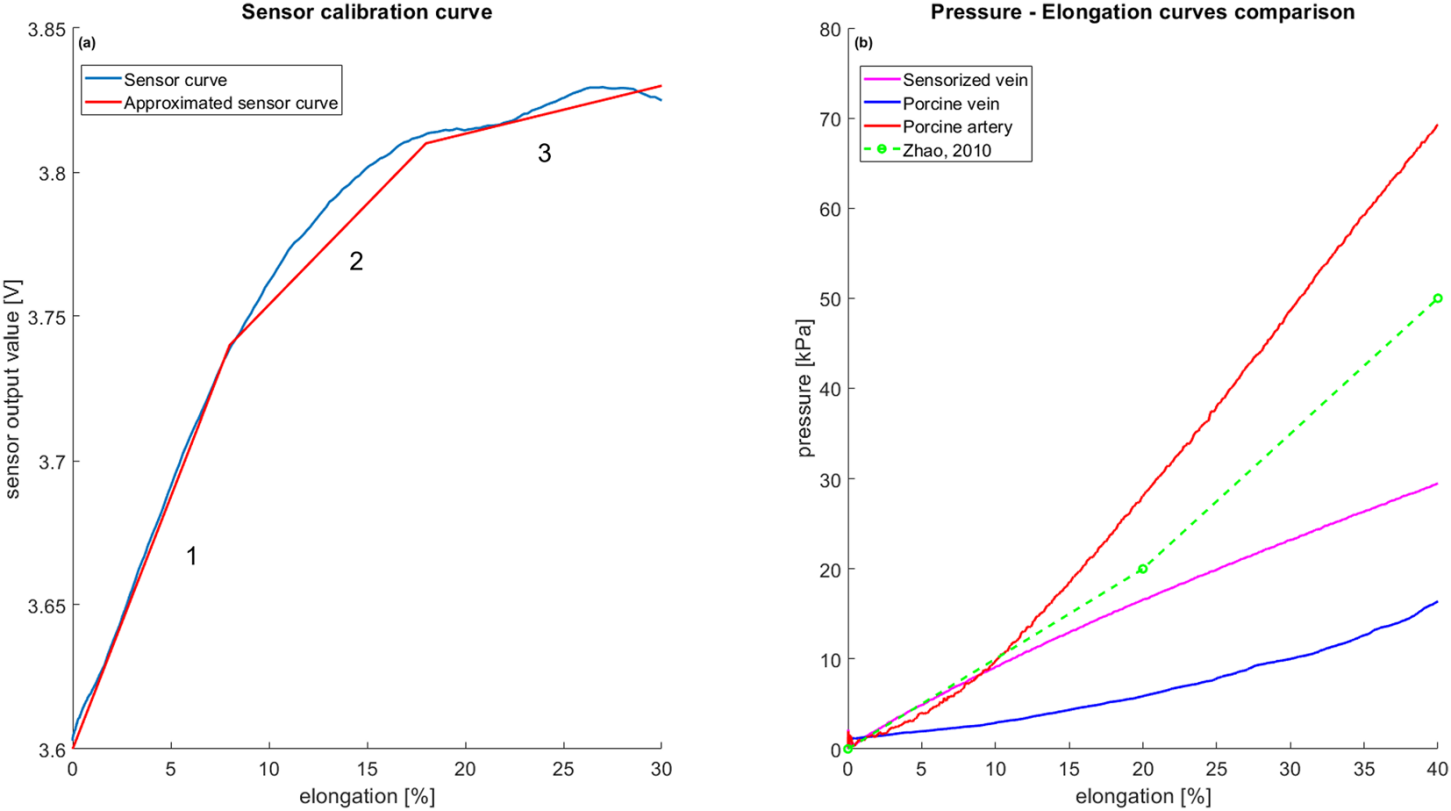
(**a**) Sensor calibration curve. Blue: the experimental curve. Red: the approximated calibration curve used in the GUI to extract vessel elongation starting from the output sensor value. (**b**) Comparison of the pressure‒elongation curves of the sensorized silicone vein (pink), porcine vein (blue), and porcine artery (red) and literature data for a porcine artery (green).

By combining the percentage of elongation applied to the sensorized vein via the Instron machine with the sensor output value recorded by the electronic circuit, the sensor calibration curve was obtained (Fig. 6a). The calibration curve allows us to obtain the percentage of deformation with respect to the sensor output value. An approximated calibration curve composed of three consecutive linear equations (Root Mean Square Error (RSME): RSME1 = 0.0033, RMSE2 = 0.0101, and RMSE3 = 0.0030) was used in the GUI (Fig. 4a) to compute and display the real-time elongation percentage, instead of sensor output, to the surgeon, and in the data analysis performed to assess the construct validity of the simulator. Furthermore, the GUI features a calibration button for the embedded sensor. Calibration should be performed at the beginning of each training session when the vessel is positioned on the simulator. The GUI enables the activation of two feedback options (acoustic and visual, using a buzzer and three LEDs) and allows the measured vessel elongation data to be recorded and streamed in real time. It is designed to be used by clinicians after brief training on its functionalities, thanks to its user-friendly design.

To compare the mechanical properties of the soft sensorized vascular structure with those of human tissues, tensile tests were performed on porcine veins and arteries. Porcine tissues are very similar to adult human tissues, and the use of these tissues allows the extraction of data and parameters for high-fidelity simulator design^42^. The pressure–elongation curves are presented in Fig. 6b. The behavior of the sensorized vein curve is equal for the 90.4% to the one obtained testing the porcine vein.

Moving from vein to artery, the pressure‒elongation curve, as expected, shows higher values being the artery tissues more rigid due to its anatomical structures^43^.

With respect to the *mechanical characterization of PVA*, after analyzing the data obtained from the compression test of the sample subjected to three freeze‒thaw cycles, Young’s modulus was found to be $\left(27.34\pm 2.79\right)\text{ kPa}$. These values are consistent with those reported in the literature. Sparks et al.^30^ reported that the elastic modulus of subcutaneous adipose tissue typically ranges between $1.9$ and $31.9\text{ kPa}$.

### Validity and usability assessment

The experimental setup used during the tests performed at the IRCAD training center in Strasbourg is shown in Fig. 4c. The experimental setup consisted of a da Vinci X (master console, robotic arms, vision chart) and the surgical table where the sensorized simulator was placed. The simulator was presented to 13 surgeons who performed two repetitions of a simple retraction task without the PVA-based surrounding tissue used to familiarize with the simulator setup. The surgeons were then asked to complete two repetitions of the complete vessel isolation task (Fig. 4b).

**Figure 7:**
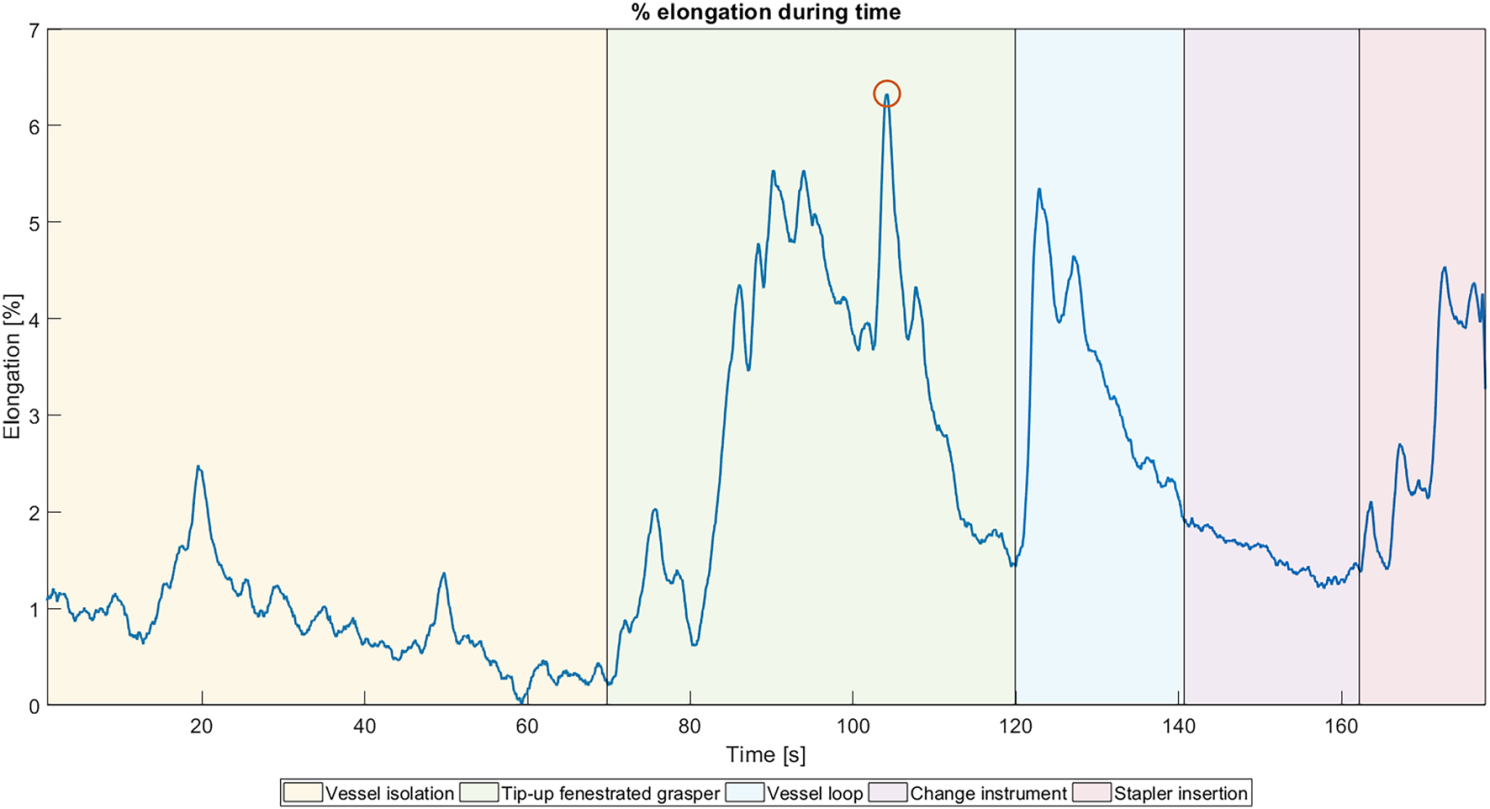
Expert elongation-signal curve recorded during task execution: percentage of sensor elongation vs. time. The different steps of the surgical procedure can be observed from the graph. From left to right: vessel isolation, insertion of the tip-up fenestrated grasper, passage of the vessel loop to lift the vein, change of the tip-up fenestrated grasper with a stapler 30, and insertion of the stapler without firing it. The red circle highlights the maximum elongation reached during the entire task.

A typical *elongation-signal* curve registered during execution of the experimental task is presented in Fig. 7. The surgeons start the procedure with the vessel isolation task, usually performed with electrocauterization of the soft surrounding tissues; then, they use a tip-up fenestrated grasper to pass under the vein and allow a vessel loop to encircle the vein itself. By using the vessel loop to lift the vein, the surgeons have sufficient space to insert the stapler. As shown in Fig. 7, the sensor data can also be used for identifying the five phases of the entire procedure: the vessel isolation step (light yellow area); the insertion of the grasper (light green area) under the vein to allow passage of the vessel loop; the passage of the vessel loop around the vein and the subsequent vessel lift (light blue area); the time needed to change the tip-up fenestrated grasper with a stapler 30 (violet area); and, finally, the insertion of the stapler without firing it (pink area).

**Figure 8:**
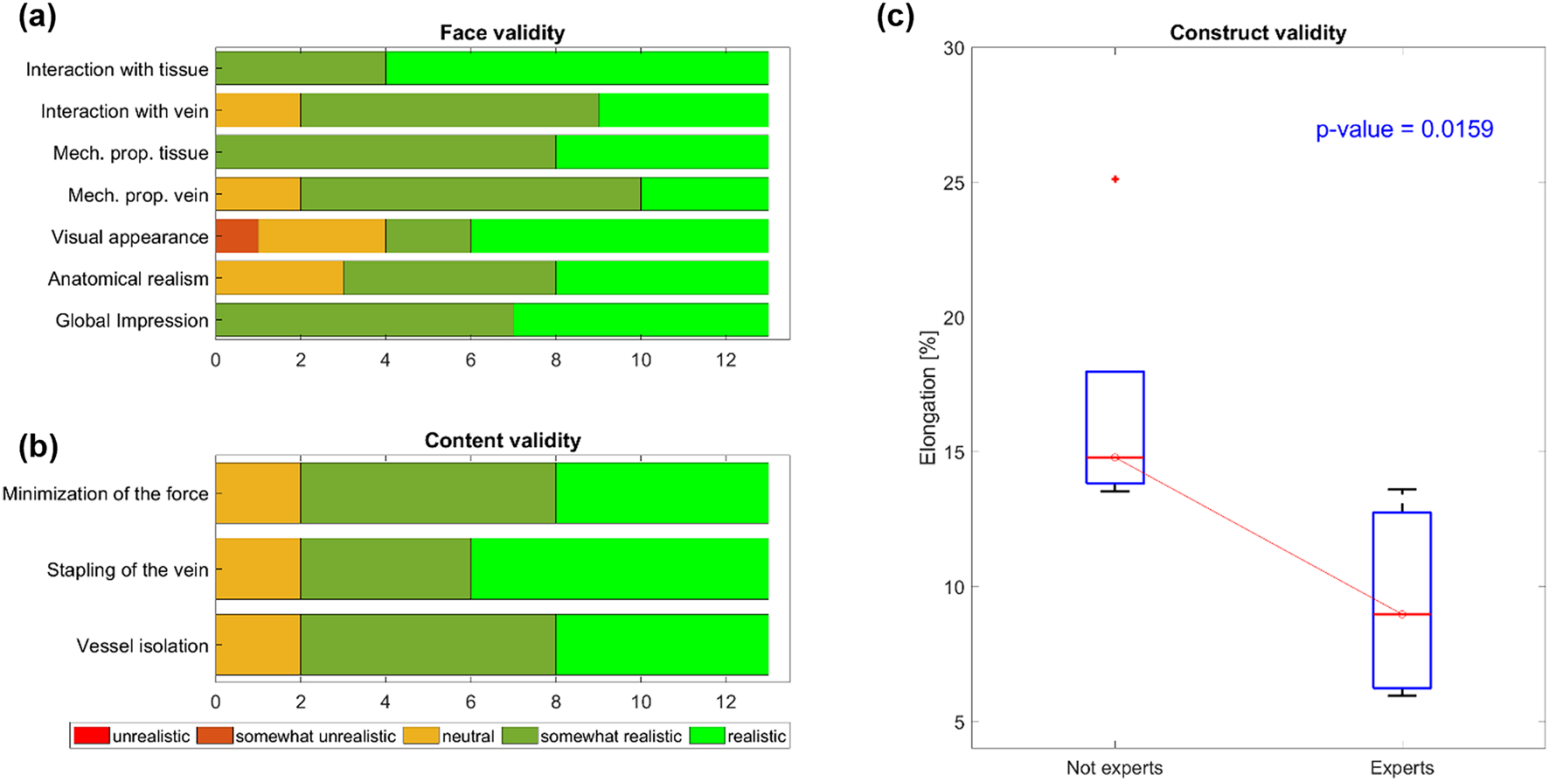
Face, content and construct validities of the simulator. (**a**) Face validity assessment of simulator realism. (**b**) Content validity assessing the capability of the simulator to teach what it is meant for. (**c**) Construct validity assessing the simulator ability to discriminate between heterogeneous levels of experience.

After performing the entire protocol, the surgeons completed the questionnaire, as described in the “Methods” section, with questions concerning both face and content validity. Construct validity was determined according to the data registered from the sensor during task execution. The results of face, content and construct validity assessments are shown in Fig. 8a–c, respectively. Regarding face validity (Fig. 8a), the simulator was considered to be realistic in terms of its overall impression. Seven surgeons considered the visual appearance of the simulator realistic, two somewhat realistic, and three neutral; only one surgeon declared the visual appearance to be somewhat unrealistic. The mechanical properties of the vein were considered realistic by three surgeons and somewhat realistic by eight surgeons, while the mechanical properties of the surrounding tissue were considered somewhat realistic or realistic by all surgeons. Considering the interaction between the tissues and the surgical instruments, seven users considered the vein to be somewhat realistic and four to be realistic; the surrounding tissue was considered to be somewhat realistic by four surgeons and realistic by nine.

Regarding content validity (Fig. 8b), the majority of the users agreed or strongly agreed on the validity of the simulator as a training tool for vessel isolation, vein stapling and minimization of the applied force. When the population was reduced to only expert surgeons (no. 5), most (80%) agreed on the usefulness of the simulator as a training tool.

Regarding construct validity, three surgeons, one expert and two novices, were excluded from the evaluation due to task incompleteness. Task incompleteness was not related to the simulator but was caused by some issues with the da Vinci robot that was unable in recognizing the used training surgical instruments. Thus, the sample size used to preliminarily assess the construct validity of the simulator was ten; these surgeons were divided into two groups based on their level of expertise: five nonexperts and five experts. The simulator was able to discriminate between two heterogeneous levels of experience by showing difference between expert and not expert surgeons, as shown in Fig. 8c. A statistical comparison between expert and nonexpert surgeons revealed a significant difference (p value = 0.0159). This demonstrates that expert surgeons outperformed nonexpert surgeons in terms of the maximum strain applied to the blood vessel.

Regarding the usability of the system, the majority of the users declared they would like to use the simulator, which they considered easy to use. Only one user thought that he or she would need a technician to setup the simulator. Most of the surgeons (92%) did not consider the simulator difficult to use, and they also thought that the learning process to manage it autonomously would be fast. The participants agreed in considering the setup *plug and play,* and they found the system to be comfortable to use.

## Discussion and conclusions

The robot-assisted manipulation of delicate tissues requires practical training to preserve patient safety. Specifically, vascular resection is carried out to treat most kinds of tumors across different surgical specialties, and improper handling of vascular structures can cause severe bleeding and complications. In this work, we propose an optimized version of a high-fidelity physical simulator that we previously developed to train this task. The simulator is a compact, portable and easy-to-use system featuring a vein based on silicone and equipped with a soft stretching sensor housed within PVA-based soft surrounding tissue. The ad hoc designed stretching sensor was integrated into the vein and mechanically characterized through monoaxial tensile tests to obtain both the sensor calibration curve and its overall mechanical properties. The latter were compared with those of the porcine vein and artery, which showed 90.4% correspondence with the vein. The PVA-based surrounding tissue was manufactured according to the literature and feedback from surgeons. Then, it was characterized by means of compressive tests and showed a Young’s modulus of $27.34\pm 2.79\text{ kPa}$ consistent with the literature data for human adipose tissue^30^. In addition, a GUI was developed to collect and stream sensor elongation data and to control real-time visual and auditory feedback. Future efforts will be dedicated to implementing further useful real-time and post-test feedback, such as the amount of time above the threshold, by following clinical needs and developing more complex algorithms to assess performance quality. In fact, thanks to the sensor embedded into the vessel, it is possible to discriminate the five different surgical task phases. This ability of the sensor could be used to implement a classification algorithm able to show novices the different phases of the procedure and guide them through task accomplishment. This could be achieved by comparing the results between experts and nonexperts and providing scores and insights into their performance.

To assess face, content and construct validity, 13 surgeons with heterogeneous levels of expertise performed vessel isolation and stapling on the proposed simulator using the da Vinci surgical robot. The simulator was considered realistic or somewhat realistic from the anatomical perspective and based on the mechanical properties of the structures’ replicas by the majority of the surgeons. Haptic feedback was collected by asking each surgeon to handle the simulated vein and adipose tissue manually. Regarding their feedback about the interaction between the simulated vessel and adipose tissue and the instruments, they declared it to be somewhat realistic or realistic. In particular, the possibility of electrocauterizing the soft surrounding tissue was appreciated because it allowed them to perform the task as in a real clinical scenario. No additional feedback (i.e., light or acoustic signals in the case of high traction force) was provided to the surgeons during the task. This testing choice allowed us to perform the simulated task as in the traditional setting. The content validity of the simulator was also assessed. Most surgeons agreed on the validity of the simulator as a training tool to teach vessel isolation, vein stapling and force minimization. The evaluation of the short- and long-term training effectiveness of the simulator would require, instead, a full training program. Future efforts will be dedicated to this part of the evaluation process. Finally, construct validity was assessed since the simulator was able to statistically discriminate between two levels of experience (expert vs. nonexpert) in terms of maximum strain applied to the vessel.

Based on the obtained results, the proposed simulator represents an encouraging starting point for the development of high-fidelity simulators of delicate tissues, such as blood vessels, integrated with sensing technologies. However, further investigations need to address the training effectiveness of the proposed simulator. Indeed, including the simulator in a longer protocol (e.g., a standard training course) can pave the way toward evaluating the retention of learned skills and their transferability in standard training settings (e.g., pig models) and clinical practice. This protocol can include the activation of visual and/or acoustic feedback triggered by the data obtained from the embedded sensor, which were not considered in this work. Additionally, the possible transferability of this specific system to other surgical specialties and procedures can be addressed by modifying vessel parameters (such as length, diameter and wall thickness). Another interesting point could be the integration of the system in a virtual reality environment to obtain a hybrid simulator in which the physical part provides the same feedback as the surgeon experiences with the real tissue and measures deformation, while the virtual reality simulator allows the development of different clinical scenarios, such as intraoperative complications like bleeding, to be displayed on top.

### Supplementary Information

Supplementary Video 1.

Supplementary Information.

## Data Availability

The data collected and analyzed during the current study are available from the corresponding author upon reasonable request.
