# Supplementary material for: Design and preliminary validation of a high-fidelity vascular simulator for robot-assisted manipulation
Source: Sci Rep. 2024 Feb 27;14:4779. doi: 10.1038/s41598-024-55351-8 (PMC10899586; doi:10.1038/s41598-024-55351-8)
Supplement: Supplementary file 2 — Supplementary Information. [file 41598_2024_55351_MOESM2_ESM.pdf]

Design and preliminary validation of a high-fidelity vascular simulator for robot-assisted manipulation

Giulia Gamberini[1,2*], Sabina Maglio[2,3,4], Andrea Mariani[1,2], Alessandro Dario Mazzotta[2,3,5], Antonello Forgione[6], Jacques Marescaux[6], Franca Melfi[7], Selene Tognarelli[2,3], Arianna Menciassi[2,3]

Questionnaire about the face, content validity and the usability of the system

PERSONAL AND PROFESSIONAL INFORMATION							
**Age**	18-24 years	25-29 years	30-50 years	50+ years			I don't want to answer
**Sex**	Male	Female					I don't want to answer
**Dominant hand**	Right	Left	Both				I don't want to answer
**Educational level**	Student	Resident	specialist	professor or expert			I don't want to answer
**Specialty**	General Surgery	Urological Surgery	Thoracic Surgery	Others			I don't want to answer
**Only if Resident, Year of Specialization**	1	2	3	4	5		I don't want to answer
**Year of experience in Laparoscopic surgery**	0 years	1-3 years	3-5 years	5-10 years	10-20 years	20+ years	I don't want to answer
**Year of experience in Robotic surgery**	0 years	1-3 years	3-5 years	5-10 years	10-20 years	20+ years	I don't want to answer
**Have you ever used a simulator for surgery both laparoscopic or robotic?**	Yes, only for laparoscopic	Yes, only for robotic	Yes, both for laparoscopic and robotic	No			I don't want to answer
**If you replied yes to the previous question (even if only for a specific kind of surgery), which kind of simulator did you use?**	Virtual reality (VR) simulator	Physical simulator	Hybrid simulator				I don't want to answer
**Hours of simulation for VR simulator**	0 hours	1-12 hours	12-24 hours	1-5 days	5-10 days	10+ days	I don't want to answer
**Hours of simulation for physical simulator**	0 hours	1-12 hours	12-24 hours	1-5 days	5-10 days	10+ days	I don't want to answer

FACE VALIDITY

		(1)	(2)	(3)	(4)	(5)
		Veramente poco realistico / Very unrealistic	Un po' irrealistico / Somewhat unrealistic	Nè realistico nè non realistico/ Neither realistic nor realistic	Un po' realistico / Somewhat realistic	Veramente realistico / Very realistic
IMPRESSIONE GLOBALE / GLOBAL IMPRESSION						
1.	**Setup completo di simulazione (confrontato con simulatori usati in precedenza) / Overall simulation setup (comparing with previously used simulators, if applicable)**	☐	☐	☐	☐	☐
STRUTTURE ANATOMICHE E REALISMO VISIVO / ANATOMICAL STRUCTURE AND VISUAL REALISM						
1.	**Realismo anatomico del simulatore senza copertura con tessuto connettivo / adiposo / The anatomical realism of the all simulator without cover with the connective /adipose tissue**	☐	☐	☐	☐	☐
2.	**Realismo anatomico del simulatore con copertura con tessuto connettivo / adiposo / The anatomical realism of the all simulator with cover with the connective /adipose tissue**	☐	☐	☐	☐	☐
3.	**Apparenza Visiva della vena/ Visual Appearance of the vein**	☐	☐	☐	☐	☐
4.	**Apparenza Visiva del tessuto connettivo/ adiposo sovrastante la vena/ Visual Appearance of the connective/ adipose tissue above the vein**	☐	☐	☐	☐	☐
		(1)	(2)	(3)	(4)	(5)
		Veramente poco realistico / Very unrealistic	Un po' irrealistico / Somewhat unrealistic	Nè realistico nè non realistico/ Neither realistic nor realistic	Un po' realistico / Somewhat realistic	Veramente realistico / Very realistic
RISPOSTA MECCANICA E APTICA / MECHANICAL AND HAPTIC RESPONSE						
1.	**Vena al tatto / Haptic Feedback of the vein**	☐	☐	☐	☐	☐
2.	**Tessuto Connettivo/ Adiposo al tatto / Haptic Feedback of the Connective/Adipose tissue**	☐	☐	☐	☐	☐

**3.**	**Realismo nell'interazione dello strumento con la vena / Realism in the instrument – vein interaction**	☐	☐	☐	☐	☐
**4.**	**Realismo nell'interazione dello strumento con il tessuto connettivo (elettrocauterizzazione) / Realism in the instrument – connective tissue interaction (electrocauterization)**	☐	☐	☐	☐	☐

CONTENT VALIDITY

Per ciascuna delle domande seguenti, si prega di indicare la propria risposta su una scala da 1 a 5 tenenedo presente quanto segue: / For each of the following questions, please indicate your response on a scale from 1 to 5, considering what follows:

(1)	(2)	(3)	(4)	(5)
Fortemente in DISACCORDO /Strongly disagree	In DISACCORDO / Disagree	NEUTRALE / Neutral	D'ACCORDO / Agree	Fortemente D'ACCORDO / Strongly agree

1. Il simulatore è utile nell'insegnare l'isolamento di una struttura vascolare / The simulator is useful in teaching vascular structure isolation:

	1	2	3	4	5	
Fortemente in DISACCORDO /Strongly disagree	☐	☐	☐	☐	☐	Fortemente D'ACCORDO / Strongly agree

2. Il simulatore è utile nell'insegnare lo stapling di una struttura vascolare / The simulator is useful in teaching the stapling of a vascular structure:

	1	2	3	4	5	
Fortemente in DISACCORDO /Strongly disagree	☐	☐	☐	☐	☐	Fortemente D'ACCORDO / Strongly agree

3. Il simulatore è utile nell'insegnare a minimizzare le forze applicate a una struttura vascolare. / The simulator is useful in teaching to minimize the forces applied to vascular structures:

	1	2	3	4	5	
Fortemente in DISACCORDO /Strongly disagree	☐	☐	☐	☐	☐	Fortemente D'ACCORDO / Strongly agree

SYSTEM USABILITY SCALE (SUS)

1. Penso mi piacerebbe utilizzare questa piattaforma per il training. / I think I would like to use this platform for training

	1	2	3	4	5	

Fortemente in DISACCORDO /Strongly disagree	☐	☐	☐	☐	☐	Fortemente D'ACCORDO / Strongly agree

2. Ho trovato il funzionamento del simulatore (setup e interfaccia utente) più complesso di quanto mi aspettassi. / I found the functioning of the simulator (setup and graphica user interface) more complex that what I was thinking.

	1	2	3	4	5	
Fortemente in DISACCORDO /Strongly disagree	☐	☐	☐	☐	☐	Fortemente D'ACCORDO / Strongly agree

3. Penso che il simulatore sia facile da usare. / I think the simulator is easy to use.

	1	2	3	4	5	
Fortemente in DISACCORDO /Strongly disagree	☐	☐	☐	☐	☐	Fortemente D'ACCORDO / Strongly agree

4. mi servirebbe il supporto di un tecnico per essere in grado di utilizzare il simulatore. / I think I will need a technical person to support me to be able to use the simulator.

	1	2	3	4	5	
Fortemente in DISACCORDO /Strongly disagree	☐	☐	☐	☐	☐	Fortemente D'ACCORDO / Strongly agree

5. Ho trovato le funzionalità dell'interfaccia utente comprensibili e chiare. / I found the interface functionalities understandable and clear.

	1	2	3	4	5	
Fortemente in DISACCORDO /Strongly disagree	☐	☐	☐	☐	☐	Fortemente D'ACCORDO / Strongly agree

6. Ho trovato difficoltà nel capire i collegamenti da fare per montare il simulatore e poterlo quindi utilizzare. / I found difficulties in understanding the physical links to setup and use the simulator.

	1	2	3	4	5	
Fortemente in DISACCORDO /Strongly disagree	☐	☐	☐	☐	☐	Fortemente D'ACCORDO / Strongly agree

7. Immagino che la maggior parte dei miei colleghi imparerebbe ad utilizzare il simulatore molto velocemente. / I image that most of my colleagues will learn how to use the simulator very fast.

	1	2	3	4	5	
Fortemente in DISACCORDO	☐	☐	☐	☐	☐	Fortemente D'ACCORDO / Strongly agree

/Strongly disagree						

8. Ho trovato il sistema intuitivo e ”plug and play”. / I found the system intuitive and “plug and play”

	1	2	3	4	5	
Fortemente in DISACCORDO /Strongly disagree	☐	☐	☐	☐	☐	Fortemente D’ACCORDO / Strongly agree

9. Mi sono sentito/a a mio agio nell’uso del simulatore./ I was comfortable with the use of the simulator.

	1	2	3	4	5	
Fortemente in DISACCORDO /Strongly disagree	☐	☐	☐	☐	☐	Fortemente D’ACCORDO / Strongly agree

10. Penso sia necessario più tempo per familiarizzare con il setup del simulatore. / I think more time is need to get familiar with the setup of the simulator.

	1	2	3	4	5	
Fortemente in DISACCORDO /Strongly disagree	☐	☐	☐	☐	☐	Fortemente D’ACCORDO / Strongly agree
